# Synchronous Pulmonary and Cecal High-Grade Neuroendocrine Carcinomas Presenting as Hepatic Metastases: A Diagnostic Challenges and Literature Review

**DOI:** 10.3390/diagnostics15192535

**Published:** 2025-10-08

**Authors:** Georgiana Elena Sârbu, Alina Ecaterina Jucan, Claudiu Vasile Mihai, Carmen Atodiresei, Madalina Ene, Carmen Ungureanu, Ioana Ruxandra Mihai, Otilia Nedelciuc, Mihaela Dranga, Cristina Cijevschi Prelipcean, Catalina Mihai

**Affiliations:** 1 Grigore T. Popa University of Medicine and Pharmacy, Strada Universității 16, 700115 Iasi, Romania; ciobanu.georgiana-elena@d.umfiasi.ro (G.E.S.); mihai_vasile-claudiu@d.umfiasi.ro (C.V.M.); carmen.ungureanu@umfiasi.ro (C.U.); ioana-ruxandra_mihai@umfiasi.ro (I.R.M.); otilianedelciuc@yahoo.com (O.N.); mihaela_dra@yahoo.com (M.D.); catalina.mihai@umfiasi.ro (C.M.); 2 Department of Gastroenterology, Saint Spiridon County Hospital, 700111 Iasi, Romania; carmen_atodiresei@yahoo.com (C.A.); cristina.cijevschi.prelipcean@umfiasi.ro (C.C.P.); 3 Department of Radiology, Saint Spiridon County Hospital, 700111 Iasi, Romania; 4 Department of Pathology, Saint Spiridon County Hospital, 700111 Iasi, Romania; 5 Rheumatology Clinic, Clinical Rehabilitation Hospital, 700661 Iasi, Romania

**Keywords:** neuroendocrine neoplasms, hepatic metastases, contrast-enhanced ultrasonography, cecal biopsy, liver biopsy

## Abstract

Background and Clinical Significance: Neuroendocrine neoplasms (NENs) are a group of malignancies that may remain clinically silent for many years. The presence of hepatic metastases can be the first clue leading to diagnosis. Case Presentation: We report the case of a 67-year-old man with intermittent tiredness and suspicious hepatic nodules detected on routine abdominal ultrasound. Contrast-enhanced ultrasonography showed arterial hyperenhancement with early washout, suggestive of metastases. Synchronous high-grade neuroendocrine carcinomas (NECs) of the lung and cecum were identified. Although the liver lesions were initially presumed to arise from the cecal tumor, liver biopsy immunohistochemistry was TTF-1 positive/CDX2 negative, whereas the cecal lesion was TTF-1 negative/CDX2 positive. This mutually exclusive immunophenotype confirmed two separate primary carcinomas. Given the high-grade histology, the patient received platinum-based chemotherapy and achieved a partial response. Conclusions: This case illustrates the diagnostic complexity of synchronous lesions and highlights the “mirage of the first lesion” phenomenon, in which the initially detected tumor may not represent the true primary site. A comprehensive, multidisciplinary strategy is crucial for establishing the correct diagnosis and guiding optimal management.

## 1. Introduction

Neuroendocrine neoplasms (NENs) represent a heterogeneous group of malignancies arising from neuroendocrine cells dispersed throughout the body [1]. The most common sites of NENs include the gastrointestinal tract, pancreas, and lungs [2,3]. According to the 2022 World Health Organization classification, NENs are fundamentally divided into two main families based on their histological differentiation: well-differentiated neuroendocrine tumors (NETs) and poorly differentiated neuroendocrine carcinomas (NECs) [4]. This distinction is of paramount clinical importance, as it reflects vastly different biological behaviors; while NETs are often characterized by an indolent course, NECs are defined by their highly aggressive nature and poor prognosis.

Our paper presents the case of a patient in whom, during the investigation of the etiology of hepatic metastases, two primary neuroendocrine carcinomas with different locations were discovered: one in the lung and the other in the cecum.

## 2. Case Presentation

**Figure 1:**
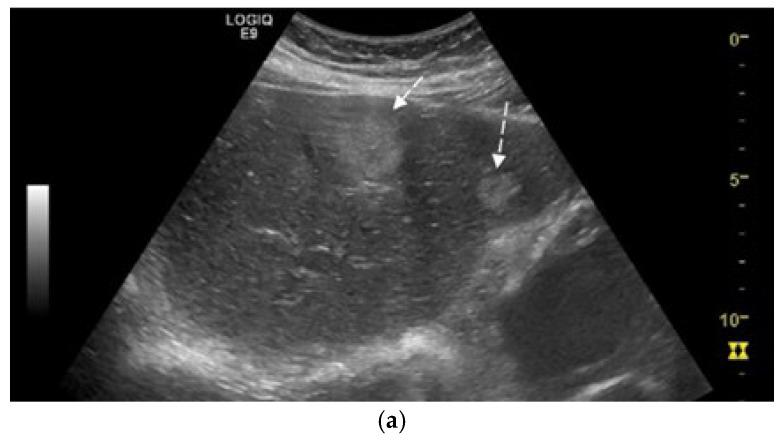

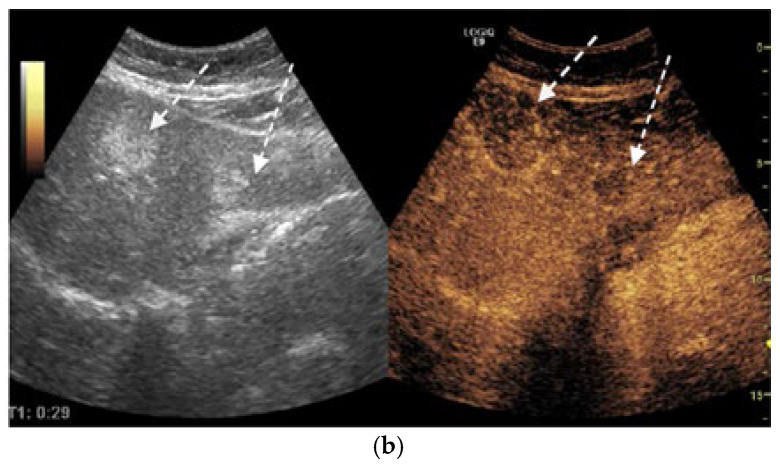
(**a**) Abdominal ultrasound B-mode image capturing two hyperechoic nodules in left liver lobe (segment IVa and III) with heterogeneous, irregular contours (white arrows). (**b**) Abdominal ultrasound in CEUS mode at the end of the arterial phase demonstrates earlier contrast washout of both nodules compared to the rest of the liver parenchyma (white arrows).

We report the case of a 67-year-old man, presented with intermittent tiredness for about three months, without associated weight loss, fever, or jaundice. The symptom was mild and episodic, occurring several times per week, and not linked to exertion or other systemic complaints. He denied abdominal pain, gastrointestinal bleeding, changes in bowel habits, cough, or flushing. From past medical history we mention chronic hepatitis C virus infection, diagnosed eight years earlier (with liver stiffness 12 kPa on transient elastography). He achieved sustained virologic response (SVR) after treatment with direct-acting antivirals (Sofosbuvir–Ledipasvir). His history also included gastric resection with gastroduodenal anastomosis for peptic ulcer disease 23 years ago, and endoscopic resection of a sessile polyp in the descending colon four years ago, confirmed as a low-grade adenomatous polyp. At the time of presentation, the patient was alert and hemodynamically stable, with vital signs within normal ranges. Physical examination revealed no jaundice, no hepatosplenomegaly, no ascites, or palpable abdominal mass, and cardiopulmonary and neurologic examinations showed no abnormalities. Laboratory investigations were within normal limits, including normal hepatic and renal function. Tumor marker levels—alpha-fetoprotein (AFP), carbohydrate antigen 19-9 (CA 19-9), and carcinoembryonic antigen (CEA)—were also within normal ranges. Abdominal ultrasound revealed three hyperechoic hepatic nodules: the largest, measuring 30 mm, in segment IVa (Figure 1a), with additional lesions in segment III (left lobe) and segment VIII (right lobe). Contrast-enhanced ultrasonography (CEUS) was performed using sulfur hexafluoride microbubbles (SonoVue^®^), administered as an intravenous bolus of 2.4 mL followed by a 5 mL saline flush. Arterial-phase enhancement of the hepatic nodules was observed at 10 s, with very early washout occurring at 29 s post-injection. This pattern is suggestive of malignant formations, most likely liver metastases (Figure 1b).

**Figure 2:**
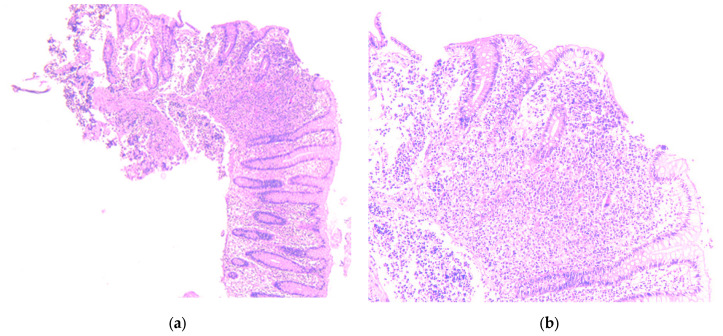
Fragment of cecum mucosa, hematoxylin-eosin staining. (**a**) 5×—the tumor distorting the normal glandular structure; (**b**) 10×—the tumor exceeds the muscularis mucosae and invades the submucosa.

**Figure 3:**
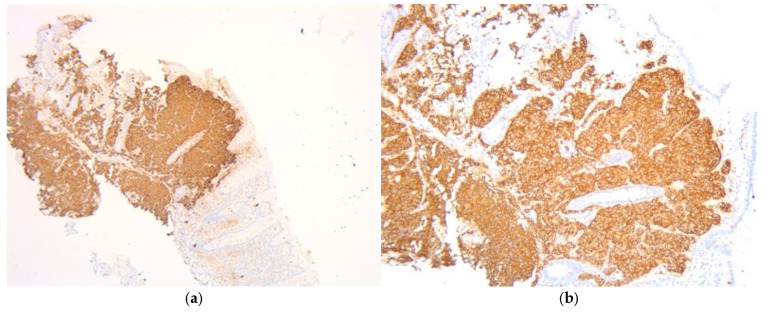
Fragment of cecum mucosa. (**a**) Chromogranin (5×) intensely and diffusely positive cytoplasmic at the level of tumor cells. (**b**) Synaptophysin (10×) intensely and diffusely positive cytoplasmic at the level of tumor cells.

**Figure 4:**
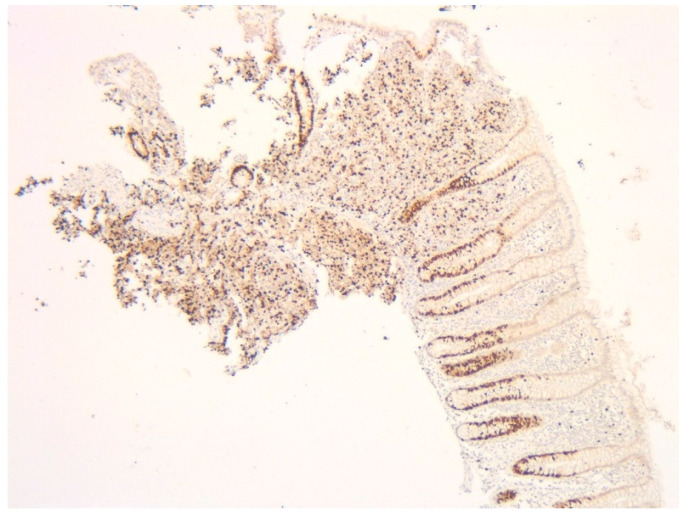
Fragment of cecum mucosa. Immunohistochemical stain for Ki-67 (5×) shows >30% positive staining in the tumor cell nuclei.

Given the high suspicion of malignancy, the patient has been scheduled for a thoraco-abdominal computed tomography (CT) scan. In the meantime, additional investigations—including esophagogastroduodenoscopy (EGD) and lower gastrointestinal endoscopy (LGE)—were performed to identify the primary source of the liver metastases. EGD showed no significant changes, with a normal gastric stump, while LGE revealed a mucosa with a normal appearance up to the ileocecal valve. A small sessile polyp, measuring 8 mm in diameter, was identified at the level of the cecum and was removed through endoscopic polypectomy. Histopathological analysis of the resected cecal polyp revealed fragments of mucosa infiltrated by a cellular proliferation with a predominantly solid architecture, extending into the submucosa. The tumor cells were monomorphic with scant cytoplasm and hyperchromatic nuclei. Critically, the mitotic rate was high, formally reported as >20 mitoses per 10 high-power fields (HPF) (Figure 2a,b). Immunohistochemical staining was positive for chromogranin (Figure 3a) and synaptophysin (Figure 3b) as well as for B-cell lymphoma 2 protein (BCL-2), and was focally positive for p53. The Ki-67 proliferation index was >30% (Figure 4). These features led to a definitive diagnosis of small cell neuroendocrine carcinoma of the cecum.

**Figure 5:**
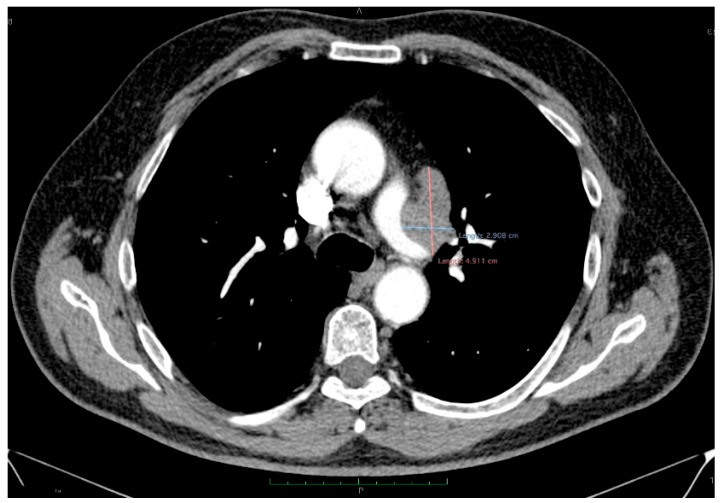
Contrast-enhanced thoracic CT (axial, arterial phase; mediastinal window) demonstrates a lobulated mediastinal mass with heterogeneous enhancement, abutting the right pulmonary artery, measuring approximately 49 × 29 mm in the aortopulmonary window.

The thoracic contrast-enhanced CT scan provided a para-aortic mediastinal tumor mass with extension into the aortopulmonary window, raising further questions about the nature and origin of the hepatic and cecal lesions. The mediastinal mass was solid, heterogeneous, irregular, lobulated, and exobronchial, measuring 50× 30 × 45 mm. Additionally, lymphadenopathies were identified in the right inferior paratracheal, subcarinal, and paraesophageal regions, as well as adjacent to the left inferior pulmonary vein and the left interlobar area (Figure 5).

**Figure 6:**
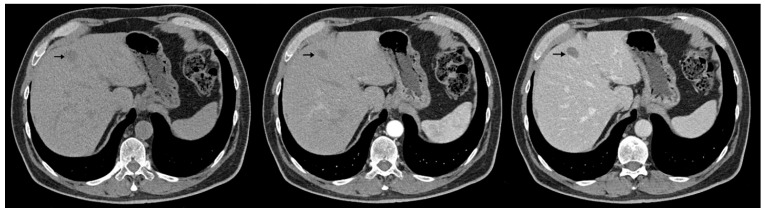
Contrast-enhanced abdominal CT (axial, non-contrast phase—left, arterial phase—center, and portal venous phase—right) shows liver sections with hypodense nodule in IVa hepatic segment exhibiting arterial-phase enhancement similar to background parenchyma and washout on the portal venous phase, measuring 1–3 cm (arrows).

**Figure 7:**
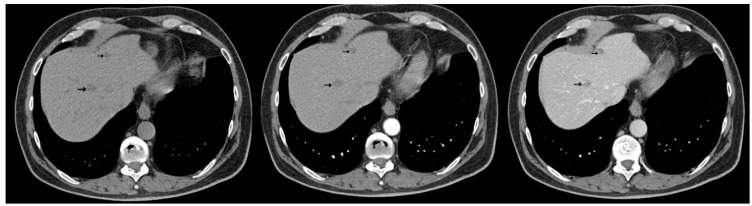
Contrast-enhanced abdominal CT (axial, non-contrast phase—left, arterial phase—center, and portal venous phase—right) demonstrates two hypodense hepatic nodules (black arrows) with a pseudocapsule appearance—more conspicuous on the portal venous phase—located in segments III and VIII, showing an enhancement pattern similar to Figure 6.

The abdominal contrast-enhanced CT scan further revealed multiple hepatic hypodense nodular lesions, measuring between 1 cm and 3 cm (Figure 6 and Figure 7).

Following this diagnosis and given the presence of the mediastinal mass and multiple liver lesions, a percutaneous liver biopsy was performed to determine the origin of the metastatic disease. Histopathological examination of the liver specimen revealed tumor infiltration with an organoid and trabecular architecture, accompanied by a prominent fibro-hyaline stroma. The report noted “evident mitotic activity” and a Ki-67 proliferation index exceeding 25%. The immunohistochemical profile was decisive, demonstrating positive labeling for synaptophysin, CK7, and TTF-1 markers, while being negative for the hepatocyte marker HepPar1 and the intestinal marker CDX2. While a specific mitotic count and necrosis status were not quantified in the available liver biopsy report, the overall morphological features, high proliferative index, and immunohistochemical profile were conclusive. They confirmed the diagnosis of liver metastasis originating from a high-grade pulmonary neuroendocrine carcinoma, distinct from the cecal primary.

Based on the definitive histological diagnosis of high-grade neuroendocrine carcinoma, the patient was referred to multidisciplinary team for management. Functional imaging with positron emission tomography (PET) was not available at the time of diagnosis, so the therapeutic strategy was based on the CT findings and histopathology.

The patient commenced first-line systemic therapy, completing four cycles of a platinum-based regimen with cisplatin and etoposide, which was well tolerated. A subsequent restaging full-body CT scan was performed for response assessment. The scan demonstrated a partial response, characterized by a 30% reduction in the size of the mediastinal lymphadenopathy conglomerate, with the multiple hepatic metastases remaining stable.

Following this favorable response, the patient proceeded with consolidation immunotherapy with the monoclonal antibody durvalumab. At the time of this report, he remains under ongoing oncologic management.

## 3. Literature Review

Neuroendocrine neoplasms (NENs) are tumors originating from neuroendocrine cells dispersed throughout the body. While relatively rare, accounting for approximately 0.5% of malignant diseases [5,6,7], their incidence has been increasing, with the gastrointestinal tract and bronchopulmonary system being the more frequent sites [8,9,10]. More than 75% of patients have metastatic disease at the time of diagnosis [11]. Identifying the primary site of NENs in the presence of liver metastases can be diagnostically challenging, particularly when more than one potential primary lesion is present.

To assess the rarity of our case, we conducted a PubMed search for case reports published in the last 10 years. The first search used the terms (“neuroendocrine neoplasm” OR “NEN”) AND (“liver metastasis”) AND (“lung” OR “pulmonary”) AND (“colon” OR “colonic” OR “cecum”) had no results. In the second search (“neuroendocrine neoplasm” OR “NEN”) AND (“liver metastasis”) AND (“lung” OR “pulmonary”) we identified 3 results but only one case described a digestive primary site (rectal NEN), with metachronous liver metastases, in a patient who also had lung adenocarcinoma [12]. The literature illustrates that liver metastases can arise from a wide array of primary NENs sites. Primary tumors are frequently located in the pancreas, small intestine (ileum, duodenum), colon, and rectum [9,10,13] while rarer origins include the esophagus and gallbladder [14,15]. Pulmonary NENs are also well-documented sources of hepatic metastases [16].

Differentiating metastatic liver from the rare occurrence of primary hepatic neuroendocrine tumors (PHNETs) is essential. A diagnosis of PHNET requires the definitive exclusion of other potential primary sites, given that the liver is a common target for metastases from extrahepatic NENs [17].

The clinical presentation of patients with NENs and liver metastases is highly variable. A significant proportion is asymptomatic, with hepatic lesions often identified incidentally during imaging studies [18]. Symptomatic patients may present with complaints like abdominal pain, weight loss, or a palpable mass [19]. In cases of functional tumors, symptoms relate to hormone overproduction. For example, metastatic carcinoid tumors can cause carcinoid heart disease when vasoactive substances like serotonin bypass hepatic metabolism due to metastases, leading to right-sided heart failure [20].

To contextualize the rarity and clinical features of our case, we reviewed the literature for other reported instances of synchronous primary neuroendocrine neoplasms. While our specific combination of two high-grade carcinomas appears to be undocumented, a comparison with other synchronous NENs highlights the unique aspects of our patient’s presentation. Table 1 summarizes a selection of these cases, detailing the primary tumor sites and the presence of metastatic disease.

This comparison underscores two key features that highlight the clinical significance of our report. First, the occurrence of two synchronous, poorly differentiated, high-grade carcinomas (NECs) is exceedingly rare; the majority of published cases describe well-differentiated neuroendocrine tumors (NETs) with a more indolent biology [12,21,22,23,24,25,26]. Second, our case is notable for its presentation with widespread hepatic metastases from the outset. As shown in the table, many reported synchronous cases are discovered as localized diseases, often incidentally. The aggressive presentation in our patient is consistent with the biology of high-grade NECs and distinguishes it from the course often seen with synchronous NETs. Therefore, this case adds valuable information to the limited literature on the presentation and management of this rare and aggressive dual malignancy.

**Figure 8:**
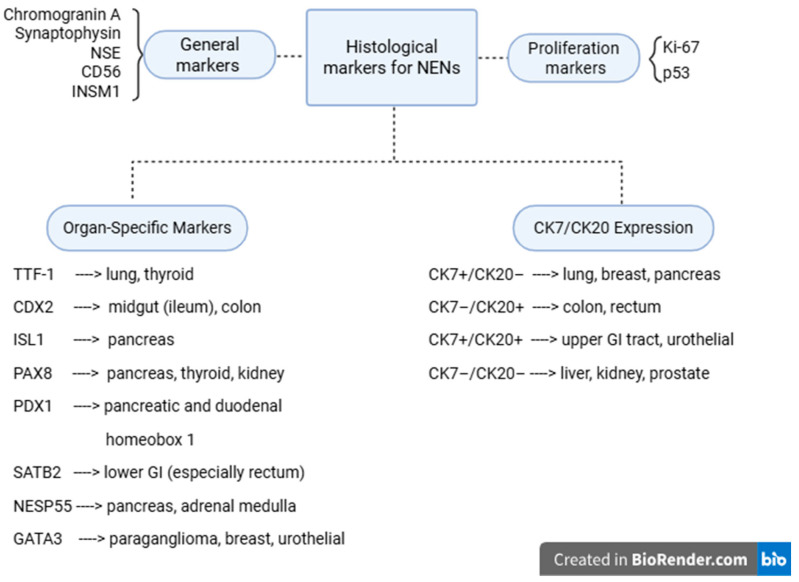
Histological markers for NENs. NSE-neuron-specific enolase, INSM1-insulinoma-associated protein 1, TTF-1—thyroid transcription factor-1, ISL1—insulin gene enhancer protein-1, NESP55—neuroendocrine secretory protein-55.

Immunohistochemistry also plays a vital role in determining the primary origin of NENs, especially in cases with liver metastases and more than one suspected primary. Organ-specific markers include TTF-1 for pulmonary or thyroid origin, CDX2 and SATB2 for intestinal primaries, PAX8 for pancreas, thyroid, and kidney, PDX1 for pancreas and duodenum, ISL1 for pancreatic NENs [27,28]. Additionally, CK7/CK20 expression patterns can aid in the localization of the tumor, where combinations such as CK7+/CK20− suggest a pulmonary or pancreatic origin, and CK7−/CK20+ are more typical of colonic tumors. The detection of specific cytokines and hormone-related markers can support tumor functionality and localization (Figure 8) [29,30,31,32].

The therapeutic approach varies based on the stage of the disease. Management options for neuroendocrine liver metastases are summarized in Table 2. For local or locoregional disease, radical surgery is the preferred treatment. In cases of hepatic metastases, options such as arterial embolization, chemoembolization, or radiofrequency ablation may be considered [33,34]. Systemic chemotherapy is indicated for neuroendocrine tumors with pulmonary localization, especially in cases of rapidly progressive disease [35,36]. This may be followed by immunotherapy using monoclonal antibodies such as durvalumab, which has shown promising results in cases of double primary cancers involving the colon and lung [37].

## 4. Discussion

The presence of two distinct primary NENs—pulmonary and cecal—diagnosed in the setting of metastatic liver disease makes this case a rare diagnostic and therapeutic challenge.

The patient had a known history of chronic hepatitis C with advanced fibrosis, which complicated the initial diagnostic process. Although the patient had achieved a sustained virologic response (SVR), the risk of hepatocellular carcinoma (HCC) was still considered, especially in the absence of regular surveillance. The history of peptic ulcer disease at a relatively young age in conjunction with the synchronous high-grade neuroendocrine carcinomas, prompted a clinical investigation for multiple endocrine neoplasia type 1 (MEN1). Accordingly, a comprehensive biochemical screening was performed, which included evaluation of calcium-phosphorus metabolism, serum gastrin, and prolactin levels; all results were within normal limits. While a detailed family anamnesis for endocrine disorders was unavailable, the normal biochemical findings substantially reduce the likelihood of an underlying MEN1 syndrome in this case. This evaluation was considered crucial, as the association between gastroenteropancreatic neuroendocrine neoplasms and suggestive clinical features warrants investigation for inherited syndromes, as emphasized in the literature [41].

The clinical presentation of patients with NENs and liver metastases is highly variable. A significant proportion is asymptomatic, with hepatic lesions often identified incidentally during imaging studies [18]. Symptomatic patients may present with complaints like abdominal pain, weight loss, or a palpable mass [20]. In cases of functional tumors, symptoms relate to hormone overproduction. For example, metastatic carcinoid tumors can cause carcinoid heart disease when vasoactive substances like serotonin bypass hepatic metabolism due to the metastases, leading to right-sided heart failure [18].

Diagnosis relies on a multimodal approach, integrating serological test, morphological imaging, functional imaging with histopathological evaluation, and immunohistochemical profiling. Available serological tests for NENs include chromogranin A (CgA), serotonin, 5-hydroxyindoleacetic acid (5-HIAA), gastrin, insulin, proinsulin, C-peptide, glucagon, vasoactive intestinal peptide (VIP), pancreatic polypeptide (PP), calcitonin, adrenocorticotropic hormone (ACTH), somatostatin, and neuron-specific enolase (NSE). [18] Conventional ultrasound often shows strong echoic nodules but lacks specificity. CEUS is more valuable, often revealing a characteristic “fast forward and fast out” arterial hyperenhancement pattern [42]. Hepatic metastases from NENs typically present as hypervascular, ring-enhancing lesions during the arterial phase [17]. However, atypical presentations, such as pseudocystic metastases due to necrosis or hemorrhage, can mimic simple cysts, complicating diagnosis [16]. CT and magnetic resonance imaging (MRI) are standard imaging modalities for the detection and characterization of both primary neuroendocrine tumors and associated liver metastases. In our case, CT scan not only confirmed the presence of hepatic lesions but also identified an additional tumor site in the mediastinum. Functional evaluations are used to detect somatostatin receptor expression on tumor surfaces. Common techniques include 68Ga-DOTATATE PET, 64Cu-DOTATATE PET, 68Ga-OTATOC PET, and PET-CT with fluorodeoxyglucose for non-functional lesions [9,18]. These imaging methods are invaluable for staging, identifying the primary tumor, and guiding treatment decisions [43]. A limitation of this case report is the absence of functional nuclear medicine imaging, such as 68-Gallium-DOTATATE PET/CT and 18F-FDG-PET/CT. While these examinations are integral to the staging and characterization of many neuroendocrine neoplasms, the decision to forego them was made deliberately by the multidisciplinary team.

Histopathological evaluation is crucial for confirming the neuroendocrine nature of a neoplasm. While standard markers like synaptophysin and chromogranin A establish neuroendocrine phenotype, newer markers like INSM1 have shown superior sensitivity and specificity [44].

In our case, we acknowledge as a limitation that a precise mitotic count and necrosis status were not quantified in the available liver biopsy report, but overall histopathology and high proliferative index are fully consistent with a high-grade carcinoma and are clearly distinct from the morphology of a well-differentiated carcinoid tumor.

Management of NENs with liver metastases is complex and often requires a multidisciplinary approach. Even in the presence of metastatic disease, an aggressive treatment strategy can lead to prolonged survival [39]. While metastatic disease is generally incurable, prognosis is highly dependent on the primary site, tumor grade, and metastatic pattern. For high-grade tumors and poorly differentiated carcinomas, the course is much more aggressive. Specifically for lung NENs, the presence of bone metastases (BM) is a significant negative prognostic factor and atypical carcinoid and hypovitaminosis D were also associated with BM, with shorter overall survival in synchronous metastases. This highlights the adverse impact of bone involvement and underscores the importance of periodic bone evaluation in patients with lung NENs, particularly those with high-grade disease, to fully assess prognosis and guide management [45].

Prognosis is highly dependent on the tumor grade (Ki-67 index), the extent of liver involvement, and the feasibility of surgical resection. While metastatic disease is generally incurable, long-term survival is achievable. Historical series show a 5-year survival of around 30% for untreated patients with liver metastases, whereas aggressive surgical resection can increase this to 60–70% or higher [10]. The case literature demonstrates that even patients with synchronous liver and splenic metastases can achieve long-term survival with a persistent, aggressive, multimodal treatment plan [39]. However, high-grade tumors and poorly differentiated carcinomas have a much more aggressive course and a dismal prognosis, often similar to that of adenocarcinoma [8,9].

## 5. Conclusions

This case illustrates the diagnostic challenges associated with non-functional neuroendocrine tumors, where the absence of clinical symptoms may lead to a delayed diagnosis, often at a metastatic stage.

While liver metastases from NENs are common and well-documented, our case stands out due to the discovery of two distinct primary neuroendocrine tumors—in the lung and cecum—during the metastatic work-up. To our knowledge, this is the first documented case of synchronous pulmonary and cecal NENs diagnosed via hepatic lesions as the initial clinical clue, emphasizing the need for comprehensive imaging and histopathology in ambiguous metastatic cases.

Further research is needed to better understand the pathophysiology, progression, and prognosis, as well as to establish optimal therapeutic management for synchronous NENs.

## Figures and Tables

**Table 1:** Reported Cases of Synchronous or Metachronous NETs [12,21,22,23,24,25,26].

Reported Cases	Localization	Synchronous (S)/ Metachronous (M)	Liver Metastases
Boehm S. et al. (2003) [21]	Pancreas and Appenidix	S	absent
Tsunenari et al. (2016) [22]	Pancreas and Ileum	S	absent
Shan et al. (2017) [23]	Small intestine and Lung	S	absent
Bruera et al. (2019) [24]	Rectum and Skin	S	absent
Song In Hye et al. (2020) [25]	Larynx and Lung	M	absent
Omori Sachie et al. (2020) [26]	Rectum and Mesentery	S	present
Hamada Y et al. (2025) [12]	Lung and Rectum	M	present
Our case	Lung and Cecum	S	present

**Table 2:** Management options for neuroendocrine liver metastases.

Therapy/ Approach	Indication	Mechanism/ Strategy	Clinical Notes/ References
Surgical resection	Localized/ resectable disease	Complete excision of primary and/or metastatic lesions	Only curative option
Cytoreductive surgery	Extensive but resectable disease	Resection of ≥90% of tumor	Improve survival and control hormonal symptoms [10]
Hepatectomy	Resectable liver metastases	Segmental liver resection, often with primary tumor removal	Robotic-assisted surgery for minimally invasive procedures [11]
Transarterial embolization and chemoembolization	Unresectable hypervascular liver metastases	Embolization ± localized chemotherapy delivery via hepatic artery	Effective in cytoreduction; supports multimodal therapy [17,38]
Radiofrequency ablation	Small, isolated liver lesions	Thermal destruction of tumor tissue using needle electrode	Used when surgery is Contraindicated [39]
Somatostatin analogs (SSAs)	Functional, well-differentiated (G1/G2), receptor-positive tumors	Symptom control and antiproliferative action via somatostatin receptor binding	Octreotide, lanreotide—used in systemic or adjuvant treatment [9,39]
Chemotherapy/ Targeted therapy	High-grade (G3), poorly differentiated/ rapidly progressing	Systemic cytotoxic agents and/or targeted molecular therapy	Platinum-based regimens or everolimus/sunitinib [17,40]
Hormonal Tumor Mapping	Functional tumors with multiple lesions	Identifies dominant hormone-secreting lesion via selective arterial secretagogue injection	Enables targeted, less extensive surgery [38]

## Data Availability

The original contributions presented in the study are included in the article; further inquiries can be directed to the corresponding author.

## Abbreviations

The following abbreviations are used in this manuscript:

NENs	Neuroendocrine neoplasms
NETs	Neuroendocrine tumors
NECs	Neuroendocrine carcinomas
SVR	Sustained virologic response
APF	Alpha-fetoprotein
CA19-9	Carbohydrate antigen 19-9
CEA	Carcinoembryonic antigen
CEUS	Contrast-enhanced ultrasonography
CT	Computed tomography
EGD	Esophagogastroduodenoscopy
LGE	Lower gastrointestinal endoscopy
IHC	Immunohistochemical tests
HPF	High power fields
BCL-2	B-cell lymphoma 2 protein
HCC	Hepatocellular carcinoma
PET	Positron emission tomography
PHNETS	Primary hepatic neuroendocrine tumors
CgA	Chromogranin A
5-HIAA	5-hydroxyindoleacetic acid
VIP	Vasoactive intestinal peptide
PP	Pancreatic polypeptide
NSE	Neuron-specific enolase
INSM 1	Insulinoma-associated protein 1
TTF-1	Thyroid transcription factor-1
ISL1	Insulin gene enhancer protein-1
NESP 55	Neuroendocrine secretory protein-55
BM	Bone metastases
